# EMPEROR-Preserved: SGLT2 inhibitors breakthrough in the management of heart failure with preserved ejection fraction

**DOI:** 10.21542/gcsp.2021.17

**Published:** 2021-10-30

**Authors:** Kerolos Wagdy, Sherif Nagy

**Affiliations:** 1 Aswan Heart Centre, Division of Cardiology, Aswan, Egypt

## Abstract

**Background:** Heart failure with preserved ejection fraction (HFpEF) is a complex disease which accounts for more than half of all HF hospital admissions with high prevalence and lack of effective evidence-based management. Sodium-glucose cotransporter 2 (SGLT2) inhibitor is a new antidiabetic drug that recently gained a new role in the management of heart failure with reduced ejection fraction but its role in HFpEF had yet to be studied.

**Study and results:** EMPEROR-Preserved trial set out to evaluate the effects of SGLT2 inhibition with empagliflozin on major heart failure outcomes in patients with HFpEF. The patients were randomized in a 1:1 fashion into two groups; to receive either empagliflozin 10 mg per day (*n* = 2, 997) or placebo (*n* = 2, 991) in addition to usual therapy. Empagliflozin led to a 21% risk reduction of the composite of cardiovascular death or hospitalization for heart failure, which was mainly related to a 29% lower risk of hospitalization for heart failure rather than effect on cardiovascular death empagliflozin. The effects SGLT2 inhibitors were consistent in all patients.

**What we have learnt:** The EMPEROR-Preserved trial is the first randomized controlled trial testing the efficacy and safety of SGLT2 inhibitor (empagliflozin) in patients with HFpEF. The trial proves that SGLT2 inhibitors (empagliflozin) can significantly reduce HF hospitalization with neutral effect on cardiovascular (CV) death.

## Introduction

Heart failure (HF) is not a single pathological diagnosis, but rather represents a wide variety of pathologies. Structural and/or functional abnormalities in the heart result in elevated intracardiac pressures and/or inadequate cardiac output at rest and/or during exercise^1^. Three distinct phenotypes of HF have been identified; HF with reduced ejection fraction (HFrEF), HF with mildly reduced EF, and HF with preserved EF (HFpEF)^1^.

HFpEF is defined as: symptoms and signs of HF, with evidence of structural and/or functional cardiac abnormalities and/or raised natriuretic peptides (NPs), and with a left ventricular (LV) EF > 50%. It accounts for more than half of all HF hospital admissions with rising prevalence and a lack of effective evidence-based management.

Multiple randomized controlled trials (RCT) have been conducted with HFpEF patients, but unfortunately failed to show any significant reduction in morbidity and mortality, although some improvements have been observed in some phenotypes under the umbrella of HFpEF. These trials include; PEP-CHF (perindopril)^2^, CHARM-Preserved (candesartan)^3^, I-PRESERVE (irbesartan)^4^, TOPCAT (spironolactone)^5^, and PARAGON-HF (sacubitril/valsartan)^6^.

Sodium-glucose cotransporter 2 (SGLT2) inhibitor is a new anti-diabetic drug that reduces blood glucose levels through increased glucose excretion in the proximal renal tubule. Additionally, it increases sodium and water excretion, leading to natriuresis and diuresis. SGLT2 inhibitors have recently gained a new role in delaying and preventing heart failure in patients with type-2 diabetes mellitus (DM)^7,8^.

Moreover, the DAPA-HF and EMPEROR-Reduced trials have shown a significant reduction in morbidity and mortality with SGLT2 inhibitors (Dapagliflozin and empagliflozin) in patients with HFrEF, regardless of DM^8–10^. The role of SGLT2 inhibitors in patients with HFpEF has not yet been studied in a large RCT.

## The EMPEROR-Preserved study

The Empagliflozin Outcome Trial in Patients with Chronic Heart Failure with Preserved Ejection Fraction (EMPEROR-Preserved) trial was a randomized, multicenter, double-blinded, placebo-controlled trial that set out to evaluate the effects of SGLT2 inhibition with empagliflozin on major heart failure outcomes in patients with HFpEF^11^.

After screening 11,583 patients, the study enrolled 5,988 patients with symptomatic heart failure and preserved ejection fraction (EF > 40%). The patients were randomized in a 1:1 fashion into two groups; to receive either empagliflozin 10 mg per day (*n* = 2,997) or placebo (*n* = 2,991) in addition to usual therapy.

The patients were followed up periodically for median duration of 26.2 months for symptoms, health status (assessed with the Kansas City Cardiomyopathy Questionnaire), and adverse events. It is noteworthy that about half of patients were diabetic.

The primary composite outcome (cardiovascular death or heart failure hospitalization) was significantly lower in the empagliflozin group compared to the placebo group (415 patients; 13.8% *versus* 511 patients 17.1%, hazard ratio, 0.79; 95% CI [0.69–0.90]; *P* < 0.001). The number of patients treated with empagliflozin needed to prevent one primary outcome event was 31 (95% CI [20–69]).

**Figure 1.**
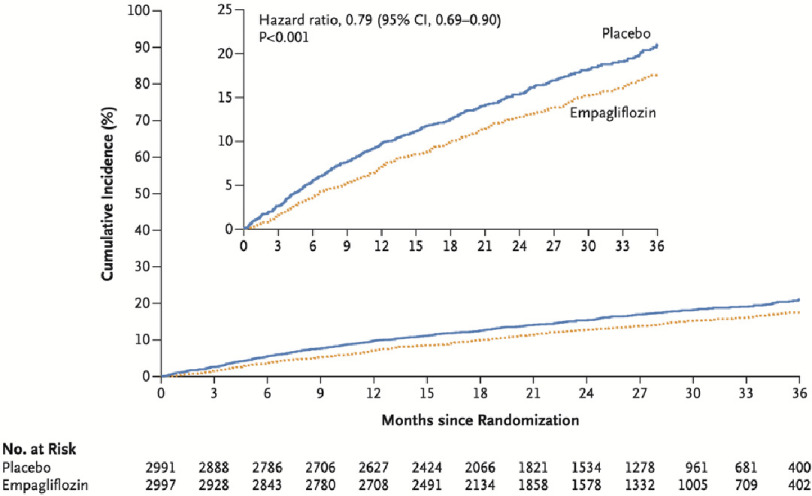
Estimated cumulative incidence of the primary outcome (composite of cardiovascular death or hospitalization for HF) in the empagliflozin group compared to the placebo group^11^.

Hospitalization for heart failure occurred in 8.6% of the empagliflozin group and 11.8% of the placebo group. However, cardiovascular death numbers were not significantly different (7.3% in empagliflozin group, *versus* 8.2% in placebo group) (Figure 1).

Regarding secondary outcomes, the total number of hospitalizations for heart failure was significantly lower with empagliflozin than with placebo (hazard ratio, 0.73; 95% CI [0.61–0.88]; *P* < 0.001) and the rate of decline in the eGFR was slower in the empagliflozin group than in the placebo group (−1.25 *vs.* −2.62 ml per minute per 1.73 m2 per year; *P* < 0.001).

## Discussion

In patients with HFpEF, empagliflozin led to a 21% risk reduction of the composite of cardiovascular death or hospitalization for heart failure, which was mainly related to a 29% lower risk of hospitalization for heart failure, rather than any substantial effect on cardiovascular death. The effects on the incidence of primary outcome events were seen consistently across all prespecified subgroups, including patients with or without diabetes.

Empagliflozin also led to a lower total number of hospitalizations for heart failure and a longer time to first hospitalization for heart failure. It is noteworthy that the percentage of patients who discontinued treatment for reasons other than death was 23% and was similar in the two treatment groups.

## What have we learned?

The EMPEROR-Preserved trial is the first randomized controlled trial testing the efficacy and safety of SGLT2 inhibitors (empagliflozin) in patients with HFpEF. The EMPEROR-Preserved trial has proven that SGLT2 inhibitors can significantly reduce HF hospitalization with neutral effect on cardiovascular (CV) death, and now the door is open for further exploration by other members of the SGLT2 family in also reducing CV mortality in HFpEF patients.
